# Monitoring the antigenic evolution of human influenza A viruses to understand how and when viruses escape from existing immunity

**DOI:** 10.1186/1756-0500-6-227

**Published:** 2013-06-11

**Authors:** Yu-Chieh Liao, Hsin-Hung Lin, Chieh-Hua Lin

**Affiliations:** 1 Division of Biostatistics and Bioinformatics, Institute of Population Health Sciences, National Health Research Institutes, Zhunan 350, Taiwan; 2 Institute of Bioinformatics and Structural Biology, National Tsing-Hua University, Hsinchu, Taiwan

## Abstract

**Background:**

The World Health Organization (WHO) organizes consultations in February and September of each year, spearheaded by an advisory group of experts to analyze influenza surveillance data generated by the WHO Global Influenza Surveillance and Response System (GISRS). The purpose of these consultations is to recommend the composition on influenza virus vaccines for the northern and southern hemispheres, respectively. The latest news of influenza viruses is made available to the public and updated on the WHO website. Although WHO discloses the manner in which it has made the recommendation, usually by considering epidemiological and clinical information to analyze the antigenic and genetic characteristics of seasonal influenza viruses, most individuals do not possess an understanding of antigenic drift and when it occurs.

**Findings:**

We have constructed a web server, named Fluctrl, and implemented a pipeline whereby HA sequence data is downloaded from the Influenza Virus Resource at NCBI along with their isolation information including isolation year and location, which are parsed and managed in MySQL database. By analyzing the frequency of each amino acid residue of the HA1 domain expressed by the viruses on annual basis, users are able to obtain evolutionary dynamics of human influenza viruses corresponding with epidemics. Users are able to upload and analyze their HA1 sequences for generating evolutionary dynamics. In addition, a distribution of amino acid residues at a particular site is represented geographically to trace the location where antigenic variants are seeded.

**Conclusions:**

Fluctrl is constructed for monitoring the antigenic evolution of human influenza A viruses. This tool is intended to inform the general public how and when influenza viruses evade the human body's immunity. Furthermore, leveraging the geographic information, the original locations of emerging influenza viruses can be traced. Fluctrl is freely accessible at http://sb.nhri.org.tw/fluctrl.

## Background

Human influenza viruses are the principal viral respiratory pathogens that cause significant human morbidity and mortality. They rapid spread around the globe, resulting in influenza epidemics and outbreaks. Vaccination is the principal way to prevent influenza and to reduce the impact of epidemics. To evade the immune response, the spike-like proteins hemagglutinin (HA) and nuramenidase (NA) on the surface of the viruses continuously mutate, which result in antigenic drift; such an event warrants a vaccine update. Unfortunately, the timely and accurate identification of vaccine strains is challenging. Therefore, WHO organizes consultations to recommend the composition of influenza virus vaccines based on influenza surveillance data; the latest news is published on its website. Although WHO discloses how to make the recommendation on vaccine composition by analyzing the antigenic and genetic characteristics of seasonal influenza viruses, most individuals do not readily understand antigenic drift and when it occurs.

Amino acid substitutions of the HA were deemed to be positively selected to reduce antibody binding and therefore were supposed to be responsible for driving antigenic drift. A great body of studies have made efforts in identifying positively selected sites for understanding antigenic evolution of human influenza A viruses [1-4]. We have previously demonstrated that the evolutionary dynamics of the positively-selected surface sites could be applied for monitoring human influenza epidemics [2]. The method we proposed is straightforward, nevertheless, it has not been utilized in any system for influenza surveillance. We therefore construct a web server named Fluctrl that implements a pipeline whereby human influenza HA viral sequences were downloaded from the NCBI database and analyzed. Users are able to freely access Fluctrl to obtain the dynamical evolutionary patterns of human influenza viruses and to trace the original locations of emerging influenza viruses.

## Implementation

### Data sources

HA protein sequences of human influenza A/H1N1, A/H1N1pdm09 and H3N2 viruses were downloaded separately from the NCBI Influenza Virus Resource [5]. For each subtype of human influenza A virus, sequences were aligned against the reference sequences, A/Puerto Rico/8/34 (YP_163735), A/California/07/2009 (ACP41953) and A/Hong Kong/1/1968 (ACC66318), respectively, by utilizing MUSCLE [6]. Duplicate sequences and those with length shorter than 267 amino acid residues were subsequently discarded. Strain information, including location and isolation year, were parsed from strain name. The aligned amino acid sequences along with the strain information were then stored in a MySQL database. The SQL tables are available for download in the website.

### HA1 evolutionary dynamics

Sequences isolated from the same year were clustered into a single group to obtain the frequency of amino acid residues at each amino acid site. For every amino acid site, if one amino acid residue reached a frequency of ≥ 0.7 during a given year, it was assigned as a single “major amino acid residue (MAA)” corresponding to that year [2]. Alternatively, multiple MAAs were assigned if more than one residue, whose frequency resided between 0.2 and 0.7, was discovered. The assembled MAAs throughout the isolation years examined represent an evolutionary dynamics perspective of human influenza viruses. The varied and white colors were used to label single and multiple MAAs, respectively. Therefore, an evolutionary dynamic pattern of HA1 proteins was clearly demonstrated. Fluctrl also allows users to upload and analyze HA1 sequences. An additional file describes the instructions for users [see Additional file 1].

### Geographic information

In an attempt to visualize the evolution of influenza viruses in the context of geography and time, we firstly employed Google Geocoding API version 3 to search the latitude and longitude coordinates of the event locations, then implemented Google Map API version 3 in Fluctrl to generate a graphical distribution of the influenza viruses within a specified period.

## Findings

### HA1 evolutionary changes reveal positively-selected sites

In our previous studies, we have identified the refined positively-selected sites by comparing avian and human influenza viruses. In addition, we have demonstrated that the evolutionary dynamics of the positively-selected sites could be applied for monitoring human influenza epidemics [2]. In an effort to escape antibody neutralization, HA proteins are expected to continuously mutate. Therefore, substitutes in an unexplored site are likely to occur and proliferate among the population, which warrants updating the list of the positively-selected sites. In this study, we have designed and built an interactive web server (called Fluctrl hereafter) enabling users to discover the positively-selected sites, thereby allowing them to monitor human influenza epidemics.

**Figure 1:**
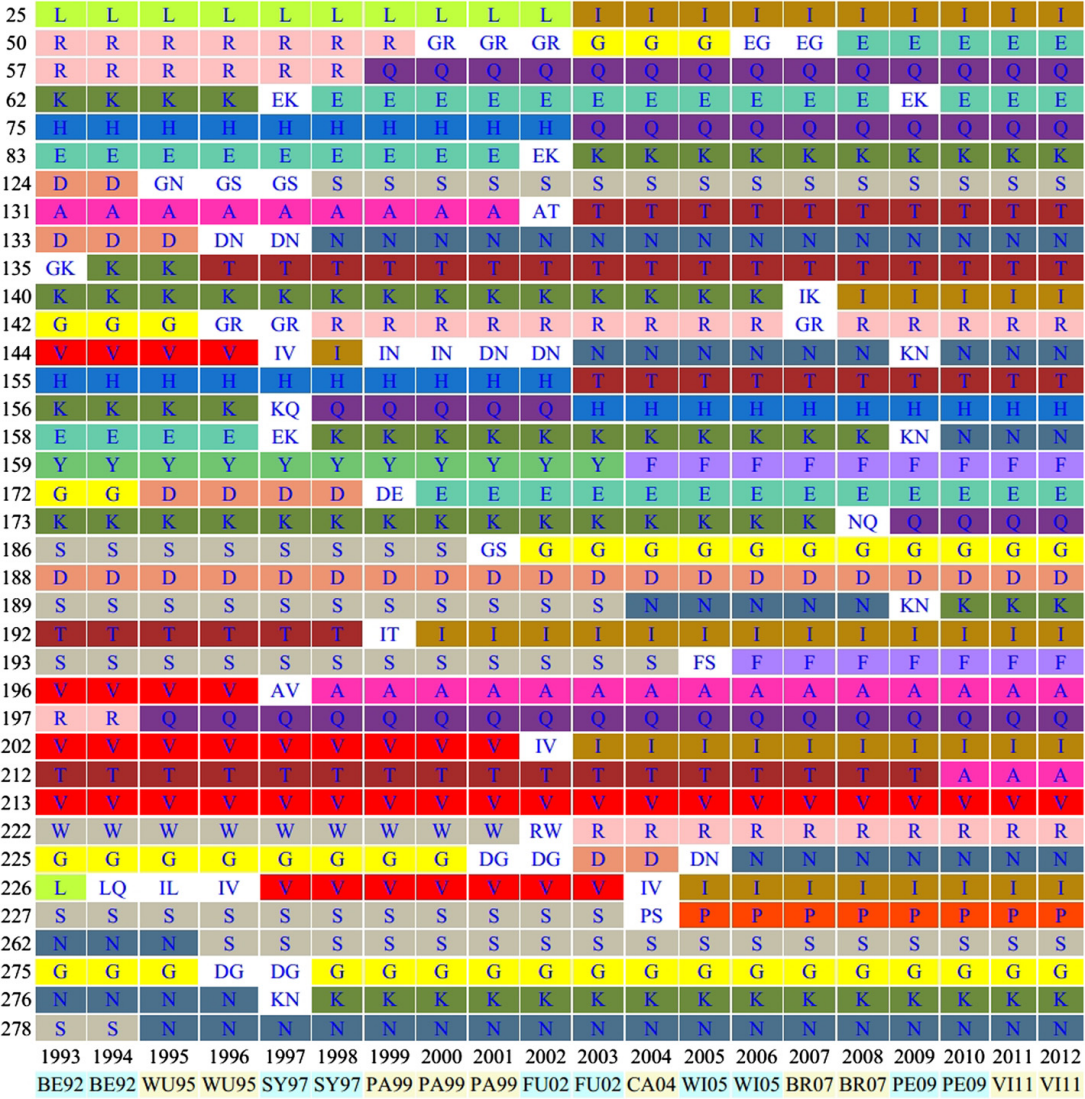
**Evolutionary dynamics of human influenza A/H3N2 viruses.** Each row represents a single amino acid site on the HA1 domain. Each column represents single or multiple major amino acid (MAA) of the site in a year. Varied colors and white were used to label the single MAA and multiple MAAs, respectively. Vaccine strains listed below the year denote annual influenza epidemics.

Based on the definition of positively-selected sites in our previous study [2], two additional sites (140 and 212) and seven additional sites (35, 82, 94, 141, 209, 267 and 274) of human influenza H3N2 and H1N1 viruses, respectively, were identified to be positively-selected after 2007. We therefore have updated the list of positively-selected sites for human influenza A/H1N1 and H3N2 viruses in the page entitled “Evolutionary dynamics”. Moreover, users are able to select any HA1 site for monitoring the evolutionary dynamics of influenza viruses in Fluctrl. Such the functionality enables users to view the antigenic evolution of human influenza viruses on any set of positively selected sites those have been identified elsewhere [1,3,4,7-9]. Figure 1 illustrates the result of an evolutionary dynamics study of human influenza A/H3N2 virus, which demonstrates that sites 140 and 212 were substituted from K to I in 2006–2008 and T to A in 2009–2010, respectively, and then remained fixed thereafter. With updated lists of positively-selected or user-defined sites, evolutionary dynamics could subsequently be applied to monitor influenza epidemics. It should be noted that, due to the limited period that the human H1N1pdm09 virus circulated after 2009, the positively-selected sites for H1N1pdm09 are adopted identical as those exhibited by the A/H1N1 virus; nevertheless, the substitutes of HA1 sites on the H1N1pdm09 virus could easily be perceived with Fluctrl. For example, to examine the evolutionary dynamics of the HA1 domain with the specific sites (all sites in the HA1 domain: 1–327), a filter was implemented with the avian H1N1 conserved sites for H1N1pdm09 virus. We discovered that five substitutes (S69T, S143G, S185T, A197T and N260D) occurred in 2012. Among the five sites, only site 69 was previously identified as a positively-selected site for the A/H1N1 viruses. We therefore suggest that the positively-selected sites for H1N1pdm09 be updated with the influenza evolution information.

### Simultaneous substitutions suggest an emerging epidemic and explain the recommended vaccinations

Influenza viruses continuously mutate as a method of escaping from antibody neutralization. Nevertheless, not all sites of HA1 were rendered selective advantages to persist in human populations. We hence identified the positively-selected sites as the sites that undergo substitutions and subsequently proliferate in the populations [1,2]. With this definition, it is not surprising that substitutions in such sites correlate with antigenic drift events, because the substitutes implicate the ability of the virus to escape from the existing herd immunity. In our previous studies, we have substantiated that three simultaneous substitutes could retrospectively reflect human influenza epidemics [2]. Since this approach is quite straightforward and easily implemented, we have analyzed the current sequence data from the NCBI Influenza Virus Resource and constructed a web server (Fluctrl) to suggest vaccine strain replacement and facilitate its selection. Although this web server mainly relies on publicly-available sequences and thus might not forecast antigenic drifts, it explains how influenza viruses evolve and why WHO recommends new vaccine formulations when a new epidemic begins to emerge. The following paragraph provides an example that explains Fluctrl’s functionality.

A northern hemisphere vaccine recommendation meeting was held by WHO in Feb. 2012 in order to make a recommended vaccine formulation containing A/California/7/2009 (H1N1)pdm09-like virus, A/Victoria/361/2011(H3N2)-like virus and B/Wisconsin/1/2010-like virus for use in the 2012–2013 influenza season. It declared that the majority of recent influenza A/H3N2 viruses were antigenically and genetically distinguishable from the vaccine virus A/Perth/16/2009 and were more closely related to A/Victoria/361/2011-like reference viruses based on influenza activity between Sep. 2009 and Jan. 2012. Accordingly, the experts suggested replacing A/Perth/16/2009 with A/Victoria/316/2011 for the new northern hemisphere season. Later, the same vaccine formulation was recommended for use in the 2013 southern hemisphere influenza season during a WHO meeting in Sep. 2012. WHO issues its recommendations and announcements publicly on its website, however, most individuals do not understand the differences between the new and former circulating viruses. In the page entitled “Evolutionary dynamics” on Fluctrl, we select “Type the specific sites” to 1–329 for the all the HA1 sites on the H3N2 virus and check the box labeled “filter with the avian conserved sites” to generate an evolutionary dynamics perspective of human influenza A/H3N2 viruses during the years between 2009 and 2012. The result exhibits the changes exhibited by human influenza A/H3N2 viruses, i.e. the substitutions of A198AS and V223IV on the HA1 domain during 2011 and 2012. The differences during this period may partly explain the genetic (or even antigenic) changes from A/Perth/16/2009-like to A/Victoria/361/2011-like viruses.

### Geographic and temporal information show the original location of antigenic variants

**Figure 2:**
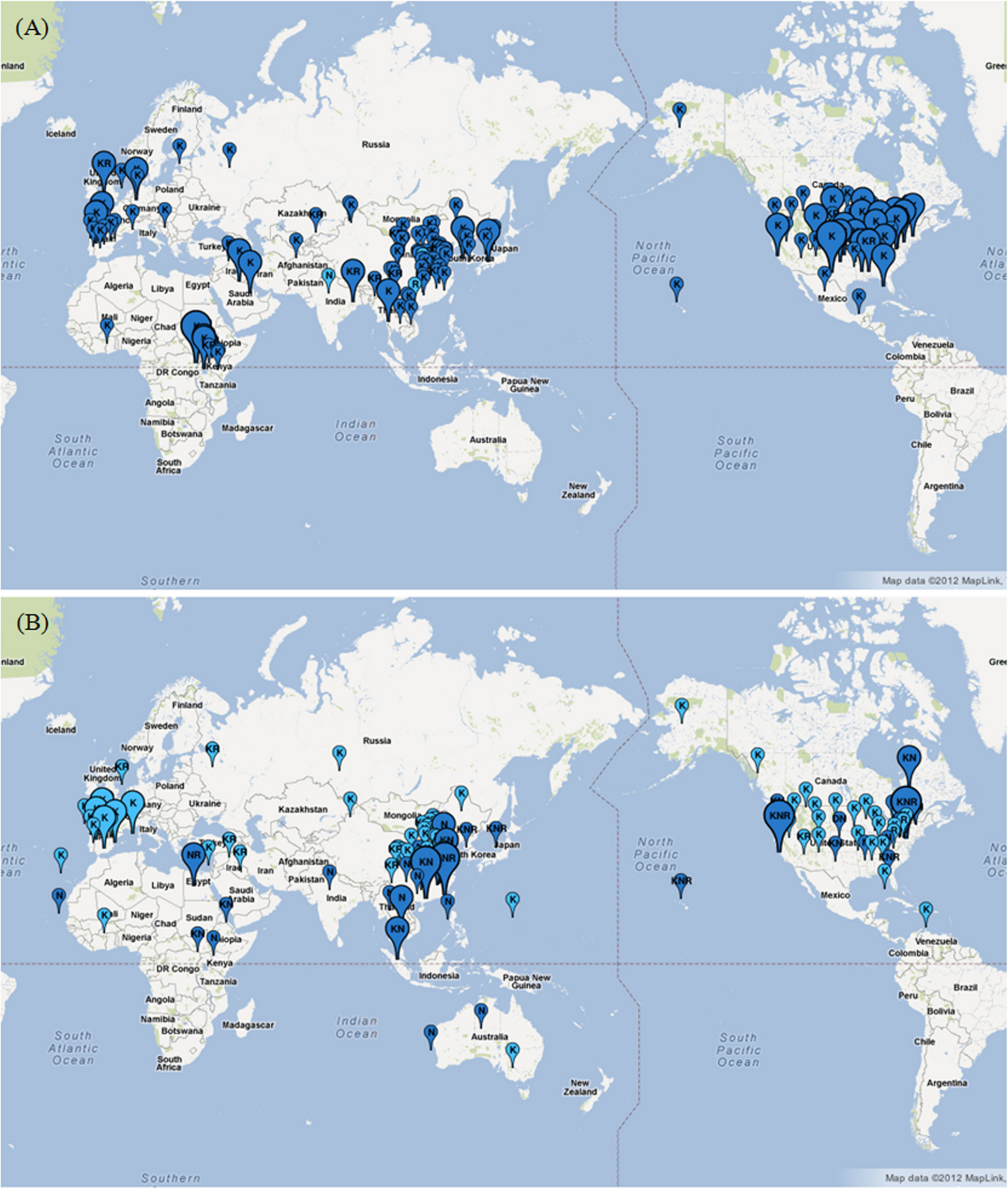
**Graphical distribution maps of human influenza A/H3N2 viruses in (A) 2008 and (B) 2009.** The balloons containing a residue exhibiting maximum frequency at the 158^th^ site are dark colored, while the other cases are light-colored. The size of the balloons are varied with the size of the sequence: small (No <=10), medium (10<No<50), and large (No>50).

In order to monitor worldwide influenza epidemics, WHO has developed a global tool (FluNet, http://www.who.int/flunet) to publically disseminate epidemiological and virological information. In addition, Flutrends (http://www.google.org/flutrends), modeling Google Search data, has been proposed to estimate flu activity data [10] and can be accessed by the public. With the above-mentioned data, users are able to ascertain the temporal and spatial dynamics of influenza activity; nevertheless, in the absence of influenza virus antigenic and genetic data, users are not able to learn how and when influenza viruses evolve to cause an emerging epidemic. To address this challenge, on the basis of publicly-available human influenza sequences, Fluctrl is able to present a timely picture of when new variants begin to emerge. Moreover, we have employed Google Map API in Fluctrl to present the geographic information of the influenza virus, with which we are able to trace the original location of the virus of interest. For example, users can leverage Fluctrl to corroborate the results that J.-R. Yang *et al.* have shown (i.e. that a new variant of influenza A/H3N2 virus emerged in East and Southeast Asia and North America in Jan. 2009, suggesting that the old vaccine composition (A/Brisbane/10/2007-like virus) be replaced with A/Perth/16/2009-like virus) [11]. We firstly examined the evolutionary dynamics of human A/H3N2 virus (Figure 1) during 2008 and 2009 to locate the substituted sites. Three simultaneous substitutions ― K158KN, NQ173Q and N189KN ― were identified and could be used to explain the emerging variant. Subsequently, we generated two maps (Figure 2) for site 158 in the page of “Geographic information” to trace where the emerging variant seeded from. By comparing the 2008 map with the 2009 version, we found that the major amino acid residue of site 158 transformed from K to NK, and those emerging N residues, located around East and Southeast Asia, coincided with the fact that new variant influenza viruses emerged from that location. The similar results could be drawn by using the sites 173 and 189.

### Surveillance within E-SE Asia facilitates vaccine strain selection

**Figure 3:**
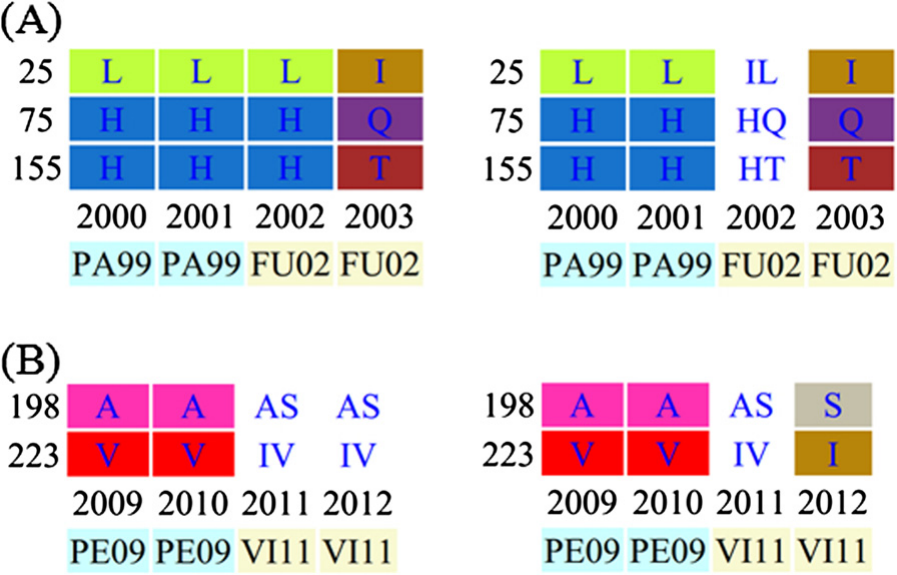
**Partial patterns of the evolutionary dynamics of regional human influenza A/H3N2 viruses.** Left and right patterns were obtained by analyzing the viruses globally and those isolated in E-SE Asia, respectively. The upper panel (**A**) reveals that the amino acid transitions occur in E-SE Asia beforehand. The bottom panel (**B**) reveals that the amino acid substitutions become fixated in E-SE Asia.

In addition to the above-mentioned evidence for the scenario ― a new viral variant emerged from East and Southeast Asia (E-SE Asia) during the 2009 season ― several studies have shown that seasonal influenza epidemics are usually seeded from E-SE Asia or China [12,13]. Fluctrl thus provides a functionality for limiting the scope of analyzed human influenza data to E-SE Asia by constraining the latitude (between −8 and 8) and the longitude (between 70 and 150) that were extracted from strain information. MAAs in an evolutionary dynamic within E-SE Asia have been observed to mutate beforehand, compared to the global dynamic, e.g. the transitions of L25I, H75Q and H155T are visible in 2002 (Figure 3A) and the substitutions of A198S and V223I become fixed in 2012 (Figure 3B). Such differences between the evolutionary dynamic patterns of the world and E-SE Asia imply that surveillance within E-SE Asia would provide accurate monitoring of the emergence of influenza variants when coupled with timely and large-scale HA sequence data. Therefore, monthly or quarterly surveillance within E-SE Asia based on routinely-performed genetic analyses of human influenza viruses would facilitate and improve vaccine strain recommendations for upcoming epidemics. Taken together, it becomes practical to detect and monitor the antigenic variants of human influenza viruses with Fluctrl on a continuous basis using the ever-increasing viral dataset to keep abreast of new influenza threats.

## Conclusions

The primary purpose of Fluctrl is to provide a user-friendly interface for individuals concerned about flu activity in order to understand the manner in which and the time when influenza viruses escape from human immunity. The web server is designed not only for influenza surveillance, but also for instructional value. With a greater amount of influenza sequences made publicly available (and promptly released), Fluctrl is a compelling platform for the detection and monitoring of human influenza variants and thus can be used to alert the general public to emerging epidemics.

## Availability and requirements

**Project name:** Fluctrl

**Project home page:**http://sb.nhri.org.tw/fluctrl

**Operation systems:** Platform independent

**Programming language:** Java

## Competing interests

The authors declare that they have no competing interests.

## Authors’ contributions

YCL conceived and designed the study. YCL, HHL, and CHL conducted the analyses. HHL and CHL built the web server. YCL wrote the manuscript. All authors read and approved the final manuscript.

## Supplementary Material

Additional file 1 Instructions for users. Click here for file
