# Supplementary material for: Monitoring the antigenic evolution of human influenza A viruses to understand how and when viruses escape from existing immunity
Source: BMC Res Notes. 2013 Jun 11;6:227. doi: 10.1186/1756-0500-6-227 (PMC3689074; doi:10.1186/1756-0500-6-227)
Supplement: Additional file 1 — Instructions for users. [file 1756-0500-6-227-S1.pdf]

We have collected HA1 protein sequences for human influenza A/H1N1, A/H1N1pdm09 and H3N2 viruses from the NCBI Influenza Virus Resource (on Jan 06, 2013). For each subtype of human influenza A virus, sequences were aligned against the reference sequences, A/Puerto Rico/8/34 (YP_163735), A/California/07/2009 (ACP41953) and A/Hong Kong/1/1968 (ACC66318), respectively by utilizing MUSCLE. Currently, 5811 H1N1, 10061 H1N1pdm09 and 12599 H3N2 HA1 viral sequences are available in Fluctrl. Each of the resulting alignments was then transformed to position-wise amino acid residues. The SQL tables are available for download. In addition, we provide the aligned HA1 sequences in FASTA format (H1N1, H1N1pdm09, H3N2, H3N2(2013)).

Evolutionary dynamics

Analyze collected sequences

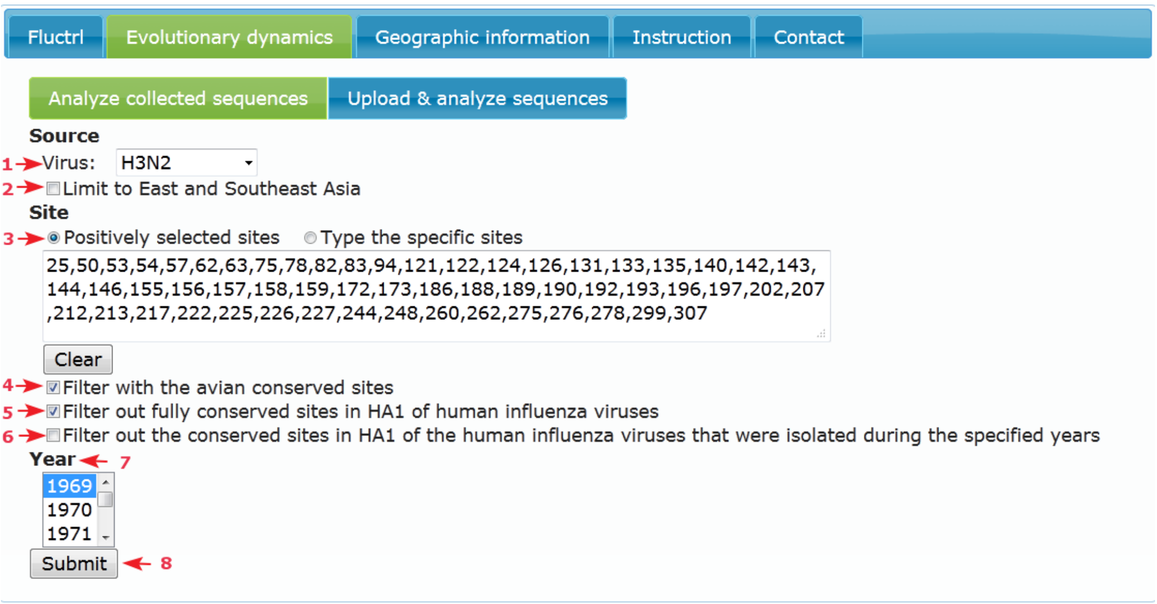

1. Select human influenza A/H1N1, H1N1pdm09 or H3N2 virus.
2. Check this box if you would like to limit the scope of analyzed sequences to East and Southeast Asia by constraining the latitude (between -8 and 8) and the longitude (between 70 and 150) of sequence location.
3. Positively selected sites: to present the evolutionary dynamics on the positively selected sites. The definition of the positively-selected sites was illustrated in [Ref. 1]. Move mouse over the check box 4, you can see the avian conserved sites.
   Alternatively, you can type any sites you would like to present in the evolutionary dynamics. (to separate by ","; to include inclusive sites by " – ", e.g., 100 – 125, 157)
4. Filter with the avian conserved site: check this box if you would like to limit the sites to the fully conserved sites in avian [Ref. 1].
5. Filter out fully conserved sites in HA1 of human influenza viruses: check this box if you would like to rule out the sites of the same MAA during all the years.
6. Filter out the conserved sites in HA1 of the human influenza viruses that were isolated during the specified years: check this box if you would like to rule out the sites of the same MAA during the specified years.

7. Year: select specific year(s) (to select multiple years by using Ctrl or Shift)
8. Submit: press submit to get the evolutionary dynamics accordingly.

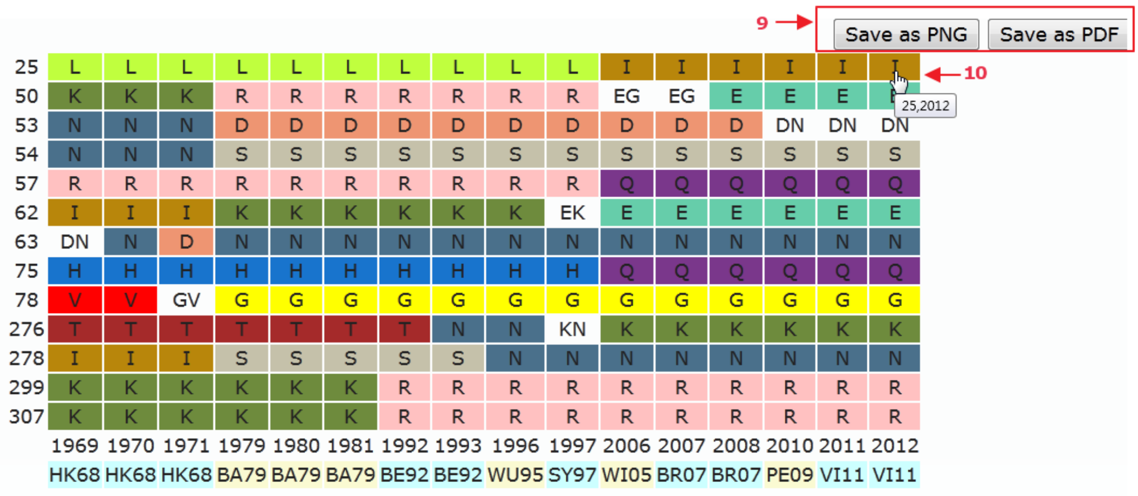

9. Save as PNG/PDF: press the bottom of Save as PNG or Save as PDF to generate the figure of evolutionary dynamics in a new window and then you can right click on it to save as PNG or PDF.
10. You can click on a major amino acid to specify site and year for generating a geographic information map which presents a graphical distribution of the site-specific residue of the influenza viruses within the specified year.

<u>Description of evolutionary dynamics result</u>

1. Row: Each row represents a single amino acid site on the HA1 domain.
2. Column: Each column represents single or multiple major amino acid (MAA) of the site in a year.
3. Color: Varied colors and white were used to label the single MAA and multiple MAAs, respectively.
4. Vaccine strains listed below the year denote annual influenza epidemics.

Upload & analyze sequences

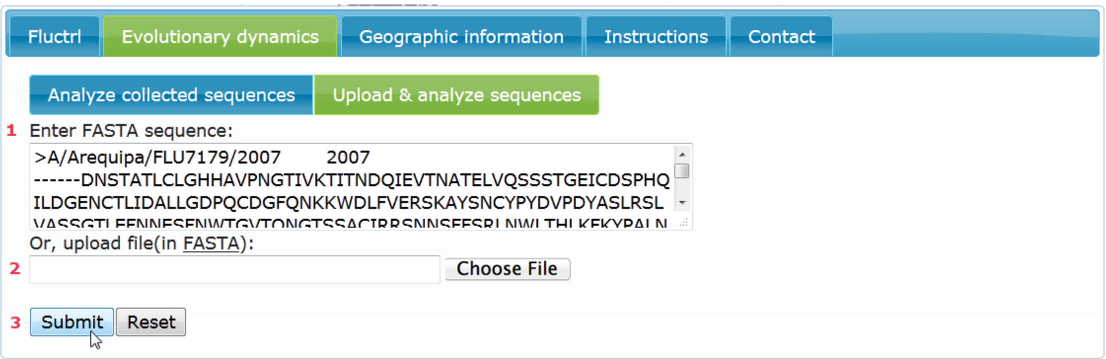

1. Enter FASTA sequence(s): paste your **<u>aligned</u>** amino acid sequences in FASTA format. Please make sure the identifier of the sequence is followed by a tab-separated year and the sequences are aligned.

An example:

>A/Puerto Rico/8/34(H1N1)   1934

DTICIGYHANNSTDTVDTVLEKNVTVTHSVNLLEDSHNGKLCRLKGIAPLQLGKCNIAGWLLGNPECDPL

LPVRSWSYIVETPNSENGICYPGDFIDYEELREQLSSVSSFERFEIFPKESSWPNHNTTKGVTAACSHAG
KSSFYRNLLWLTEKEGSYPKLKNSYVNKKGKEVLVLWGIHHPSNSKDQQNIYQNENAYVSVVTSNYNRRF
TPEIAERPKVRDQAGRMNYYWTLLKPGDTIIFEANGNLIAPRYAFALSRGFGSGIITSNASMHECNTKCQ
TPLGAINSSLPFQNIHPVTIGECPKYVRSAKLRMVTGLRNIPSIQSR

2. Or, upload file: upload a FASTA file containing aligned amino acid sequences
3. Submit: press submit to get the evolutionary dynamics accordingly.

Note: Please make sure that you have aligned your sequence file before uploading. (The lengths of aligned amino acid residues for H3N2 and H1N1(pdm09) are 329 and 327, respectively.)

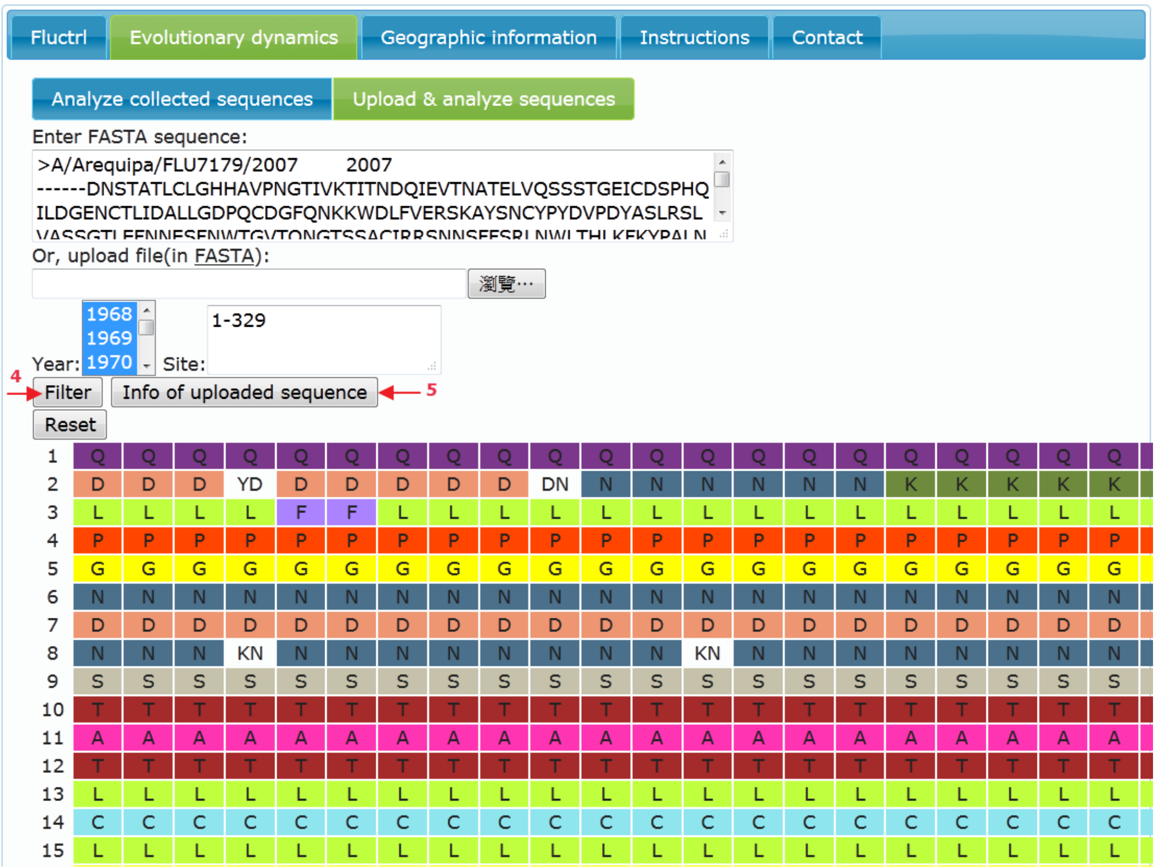

4. Filter: select specific year(s) (to select multiple years by using Ctrl or Shift) and type specific sites to generate another evolutionary dynamic in a new window.
5. Info. of uploaded sequence: press this button to see the statistics of the uploaded sequences (the results are as follows). Please check your sequence data if the number of the uploaded sequences is different from the number of sequences used for analysis.

You have uploaded 10428 sequences
10428 have been used for evolutionary dynamics

1968	1969	1970	1971	1972	1973	1974	1975	1976	1977	1978	1979	1980	1981	1982	1983	1984	1985	1986	1987	1988	1989	1990	1991	1992	1993
23	9	9	9	23	6	14	18	19	17	9	12	14	14	28	27	29	36	15	24	29	90	49	60	103	158

If you would like to upload your own sequences (for example, new circulating human influenza A/H3N2 sequences in 2013) and to compare them with the previous evolutionary dynamics, you can simply combine the two files, H3N2 and H3N2(2013), into one FASTA file. Then upload the file to fluctrl to generate an evolutionary dynamics.

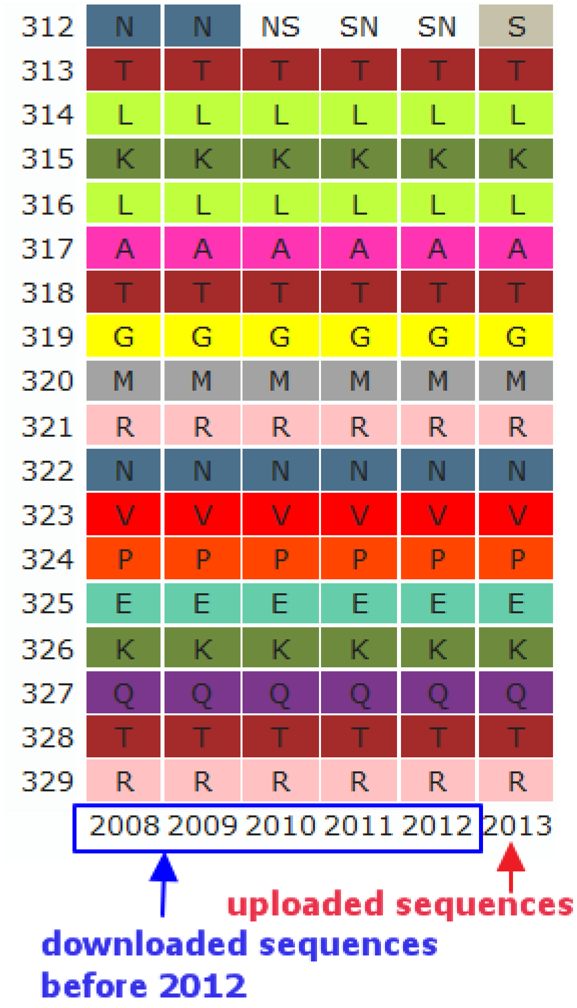

Geographic information

Single map

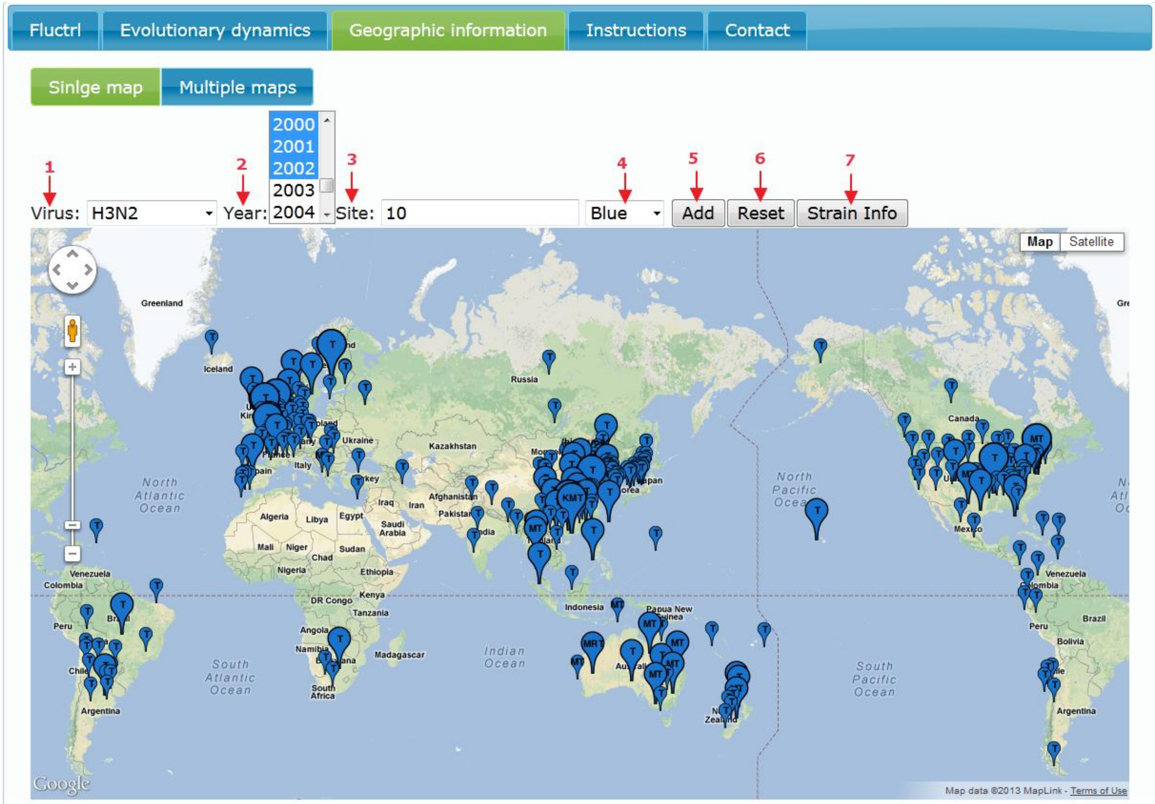

1. Virus: select human influenza A/H1N1, H1N1pdm or H3N2 virus.
2. Year: select specific year(s) (to select multiple years by using Ctrl or Shift)
3. Site: type a specific site. Any site of HA1 can be typed here, a drop-down list is optionally provided for the positively-selected sites.
4. Color: select a balloon color.
5. Add: press add button to generate a map. You can add other geographic information on the same map.
6. Reset: clear the geographic information presented in the map.
7. The information presented in the map can be showed in a new window by clicking on the button of Strain Info.

Multiple maps

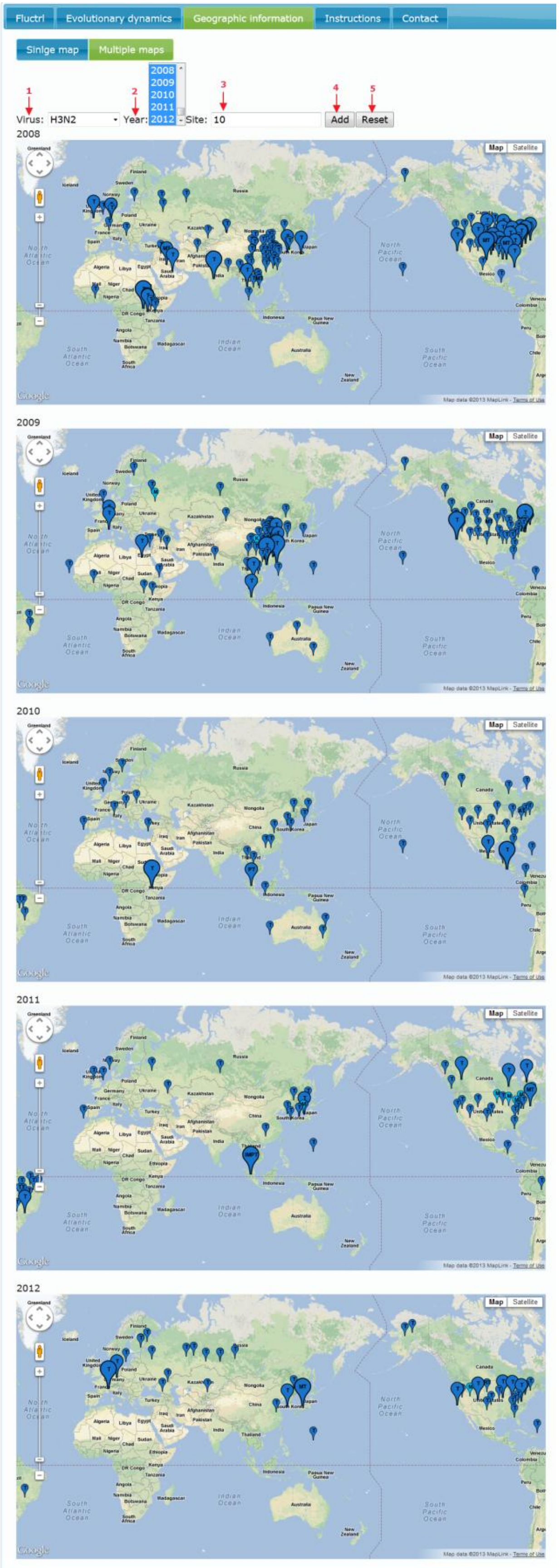

1. Virus: select human influenza A/H1N1, H1N1pdm or H3N2 virus.
2. Year: select multiple years (five years at most, to select multiple years by using Ctrl or Shift)
3. Site: type a specific site.
4. Add: press add button to generate multiple maps. The maps of the first five years are generated accordingly.
5. Reset: clear the geographic information presented in all maps.

Description of geographic map

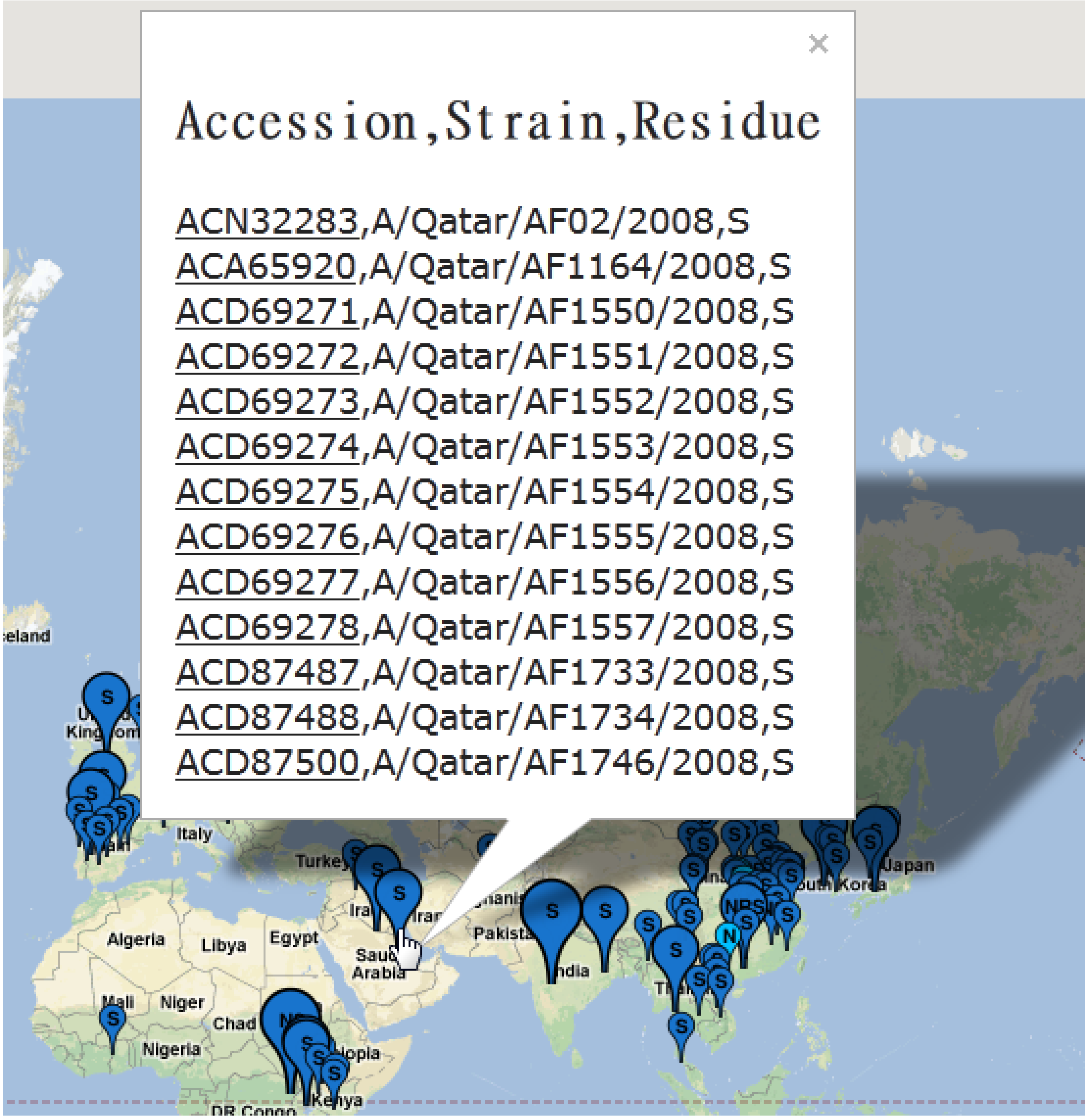

1. Balloon size: The sizes of the balloons are varied with the size of the viral sequences: small (No <=10), medium (10<No<50), and large (No>50).
2. Balloon color: The balloons containing a residue exhibiting maximum frequency (greater than or equal to 0.7) at the site are dark colored, while the other cases are light-colored.
3. Click on the balloon of the map to show the sequences information in this location, including sequence accession number, strain name and the amino acid residue of the specific site. Click on the underlined accession number to automatically link to NCBI protein database.

Reference

1. Liao, Y.C., F.C. Chen, and C.A. Hsiung, *Contrasting substitution patterns between HA proteins of avian and human influenza viruses: Implication for monitoring human influenza epidemics.* Vaccine, 2010. **28**(50): p. 7890-6.
